# Type 2 diabetes mellitus prevalence and risk scores in treated PLWHIV: a cross-sectional preliminary study

**DOI:** 10.1186/s13104-019-4183-6

**Published:** 2019-03-15

**Authors:** Sepiso K. Masenga, Paul Toloka, Kaseya Chiyenu, Ilubala Imasiku, Hope Mutengo, Oscar Ngongo Ulungu, Zangi Mallesu, Eunice Mulenga, Macwañi Mutukwa, Kingsley Kamvuma, Benson M. Hamooya

**Affiliations:** 1 School of Medicine and Health Sciences, Mulungushi University, Livingstone Campus, Livingstone, Zambia; 2 Research Section, Pathology Laboratory Department, Livingstone Central Hospital, Akapelwa Street, Livingstone, Zambia; 3 Internal Medicine, Livingstone Central Hospital, Akapelwa Street, Livingstone, Zambia; 4 Department of Biomedical Sciences, School of Health Sciences, University of Zambia, Lusaka, Zambia; 5 School of Public Health, University of Zambia, Lusaka, Zambia; 6 Chikankata College of Biomedical Sciences, Private Bag Sector 2, Mazabuka, Zambia

**Keywords:** Diabetes mellitus, Diabetes risk scores, Combination antiretroviral therapy

## Abstract

**Objective:**

This was a preliminary study whose objective was to estimate the prevalence and risk of developing type 2 diabetes mellitus (T2DM) among people living with HIV (PLWHIV) based on diabetes risk assessment scores.

**Results:**

The study was composed of 234 PLWHIV with median age (interquartile range, IQR) of 44 (36, 52) and a female preponderance of 66%. The median risk scores (IQR) for developing T2DM was 5 (2, 9). Based on the risk scores, 5% of PLWHIV were at high risk for developing T2DM close to 3.4% actual prevalence in the study population. This study demonstrated the importance of using a cheap and fast method for identifying high risk individuals for developing T2DM.

**Electronic supplementary material:**

The online version of this article (10.1186/s13104-019-4183-6) contains supplementary material, which is available to authorized users.

## Introduction

Diabetes mellitus type 2 (T2DM) with its devastating complications is increasingly becoming a challenge among people living with HIV (PLWHIV) [1–3]. The underlying causes are multifactorial and the mechanisms are still elusive though combination antiretroviral therapy (cART) [2, 4], acute systemic inflammation in HIV infection, and chronic inflammation evidenced by suboptimal persistent inflammatory markers with cART usage [5] in addition to traditional risk factors have been implicated as possible mechanisms of T2DM development. Some cART have also been implicated in the development of T2DM by causing metabolic derangements and dyslipidemia [2, 5, 6].

The prevalence and risk of diabetes among PLWHIV is increased compared to the general population [4, 7]. In developing countries, the prevalence of diabetes ranges from 2 to 14% [8]. In Sub-Saharan Africa, there is paucity of studies, however, prevalences of 1–12% have been reported [9]. Several studies have demonstrated that PLWHIV on cART do have an increased risk for developing diabetes mellitus [6, 10, 11] including research from Zambia [12] though not much has been documented locally.

Identification of individuals at risk of developing T2DM is very critical to care and patient management. The international diabetes federation (IDF) uses a screening tool [13] that estimates the risk for developing T2DM in 10 years. This tool is based on the Finnish Diabetes Risk Score (FINDRISC) developed and designed by Lindstrom and Tuomileht [14]. It is a ‘simple, fast, inexpensive, noninvasive, and reliable tool to identify individuals at high risk for developing type 2 diabetes’ [14]. Owing to the high cost of laboratory testing, this tool is warrantable for low-income countries as a way of scaling down costs without compromising standards of diagnosis. However, there are several other diabetes risk assessment tools such as QDiabetes^®^, Leicester Risk Assessment (LRA), and Cambridge Risk Score (CRS) algorithms [15]. Though, there is seemingly no agreement on the best tool, we chose the FINDRISC based assessment tool as it seemed conservative when compared to the others [15].

We conducted a preliminary study to determine the prevalence of T2DM and the significance of using diabetes risk score sheets to identify individuals who were at high risk of developing T2DM. Moreover, this study was prodded by the increasing burden of T2DM cases at Livingstone Central Hospital (LCH) by more than 10% (both HIV positive and HIV negative combined) since 2014 [16]. With no segregated data on the prevalence and risk of T2DM in HIV, this is most likely the first study known to us to document this information.

## Main text

### Methods

#### Study design and site

We conducted a cross-sectional study at Livingstone Central Hospital (LCH). Livingstone Central Hospital is a referral hospital and the largest in Southern Province of Zambia.

#### Study population and setting

Currently, the ART clinic attends to about 3875 HIV positive adults. We screened a group of HIV positive individuals enrolled in the ART clinic at LCH for T2DM and also reviewed additional files from the ART clinic research data base. This data base was created contemporally with participant enrolment for further research.

#### Study period

This study was conducted between April and July, 2018.

#### Sample size calculation

With an HIV infected population size of 3875 at LCH ART Clinic, we hypothesized a maximum percentage prevalence of 50% and 95% confidence level, and we used an online OpenEpi software [17] to compute the minimal sample size required to estimate the prevalence of diabetes among PLWHIV using the formulae below:

$${\text{Sample size }}\left( {\text{n}} \right)\, = {{\,\left[ {{\text{DEFF}}*{\text{Np}}\left( { 1- {\text{p}}} \right)} \right]} \mathord{\left/ {\vphantom {{\,\left[ {{\text{DEFF}}*{\text{Np}}\left( { 1- {\text{p}}} \right)} \right]} {\left[ {\left( {{\text{d2}}/{\text{Z21}} - \alpha / 2*\left( {{\text{N}} - 1} \right)\, + \,{\text{p}}*\left( { 1- {\text{p}}} \right)} \right)} \right]}}} \right. \kern-0pt} {\left[ {\left( {{\text{d2}}/{\text{Z21}} - \alpha / 2*\left( {{\text{N}} - 1} \right)\, + \,{\text{p}}*\left( { 1- {\text{p}}} \right)} \right)} \right]}}$$

where N is the population size; p is hypothesized % frequency of outcome factor in the population; d is confidence limits as % of 100 (absolute ±  %, which is 5%); DEFF is the design effect.

Total sample size was 350. We had two outcome variables in the study; risk scores and prevalence of T2DM and because of paucity of studies, our sample size calculation was based on prevalence. We decided to use the 350 maximum sample size. Unfortunately, we only managed to recruit 234.

#### Sampling method

We used simple random sampling to select participants who came for routine visits each day using an online random number generator. We employed the same mechanism when reviewing files in the data base.

#### Eligibility criteria

*Inclusion* We included all consenting participants who had complete information on HIV status and all variables used to calculate risk scores. Our study only included adults (18 years and above).

*Exclusion* We excluded participants who did not complete the interview, those who were sick and those coming for other medical conditions other than routine checkups.

#### Data collection and variable definitions

We used the IDF diabetes risk assessment form and the International physical activity questionnaire to collect data from PLWHIV attending routine clinical visits at LCH. Both forms are validated. Data was collected by the primary investigators. The variables and risk denomination based on score for the participants are presented as they appear in the diabetes risk assessment form (see Additional file 1). We further dichotomized our primary outcome variable risk, as ‘low’ (< 7 scores) and ‘increased’ (≥ 7 scores) as shown in Tables 1 and 3 based only on the risk scores. Therefore ‘risk’ is only to be understood in the context of risk scores as outlined in this study.

**Table 1:** Factors associated with increased risk for developing T2DM based on risk scores

Variables	N = 234	Low risk (< 7 scores), n = 135 (58%)	Increased risk (≥ 7 scores), n = 99 (42%)	p value
Age, median years (IQR)	44 (36, 52)	42 (34, 50)	48 (39, 56)	*< 0.001*
Age category (years)				
18–35	57 (24.4)	45 (33.3)	12 (12.1)	*< 0.001*
36–45	73 (32.3)	49 (36.3)	24 (24.2)	
46–55	66 (28.2)	29 (21.5)	37 (37.4)	
56–65	33 (14.1)	9 (6.7)	24 (24.2)	
66–90	5 (2.1)	3 (2.2)	2 (2.0)	
Gender, n (%)				
Female	155 (66.2)	76 (56.3)	79 (79.8)	*< 0.001*
Male	79 (33.8)	59 (43.7)	20 (20.2)	
BMI, median (IQR) kg/m^2^	22.4 (19.7, 27.1)	21.1 (18.6, 22.6)	27.3 (23.6, 31.0)	*< 0.001*
Waist circumference (cm)	80 (72, 90)	76.0 (70.0, 80.0)	92.0 (83.0, 99.5)	*< 0.001*
Duration on ART, median months (IQR), n = 233	96 (48, 132)	84 (60, 126)	96 (48, 138)	0.221
ART regimen, n = 206				
TDF/3TC/EFV	148 (71.8)	81 (69.2)	67 (75.3)	0.811
TDF/3TC/NVP	19 (9.2)	10 (8.5)	9 (10.1)	
TDF/3TC/LPV/r	15 (7.3)	9 (7.7)	6 (6.7)	
ABC/3TC/NVP	1 (0.5)	1 (0.9)	0 (0.0)	
ABC/3TC/EFV	5 (2.4)	3 (2.6)	2 (2.2)	
AZT/3TC/LPV/r	7 (3.4)	5 (4.3)	2 (2.2)	
AZT/3TC/NVP	11 (5.3)	8 (6.8)	3 (3.4)	
CD4+ absolute count *median (IQR)* cells/µl, n = 148	473 (360, 644)	435 (306, 599)	544 (398, 674)	0.345
HIV RNA viral load (copies/ml), n = 192	33 (20, 308)	31 (20, 273)	20 (20, 186)	0.724
Hypertension				
No	197 (84.2)	126 (93.3)	71 (71.7)	*< 0.001*
Yes	37 (15.8)	9 (6.7)	28 (28.3)	
Minutes of weekly vigorous activity	0 (0, 90)	0 (0, 120)	0 (0, 105)	0.234
Minutes of weekly moderate activity	60 (9, 123)	45 (0, 120)	60 (8, 120)	0.875
Minutes of weekly walking activity	60 (30, 120)	60 (30, 120)	40 (18, 70)	0.293
Days of weekly vegetable intake	7 (7, 7)	7 (7, 7)	7 (4, 7)	*0.028*
Days of weekly fruit intake	1 (0, 3)	1 (0, 3)	1 (0, 3)	0.770
Minutes spend sited every weekend	300 (120, 480)	300 (120, 375)	300 (180, 420)	0.086
Minutes spend sited every weekday	180 (120, 360)	180 (90, 303)	180 (120, 360)	0.501
Fasting blood sugar	5.2 (4.7, 5.8)	5.1 (4.7, 5.5)	5.2 (4.8, 5.9)	0.110

*n* number of participants, *%* percentage, *BMI* body mass index, *T2DM* type 2 diabetes mellitus, *IQR* interquartile range, *TDF* tenofovir disoproxil fumarate, *3TC* lamivudine, *EFV* efavirenz, *NVP* nevirapine; *LPV/r* lopinavir/ritonavir, *ABC* abacavir, *AZT* azidothymidine, also called zidovudine

p values less than 0.05 are shown in italic

#### Diagnostic criteria of T2DM

T2DM patients were classified based on history and current use of antidiabetic medicine. Diagnosis of diabetes employed in the clinic was based on elevated fasting blood sugar on more than two separate occasions with classical signs of diabetes. The IDF criteria was employed.

#### Data analysis

We used the IBM statistical package for social sciences (SPSS) version 22 for data analysis. Chi square test or fishers’ exact test was used to determine association between two categorical variables (Tables 1, 2 and 3); Column percentage is shown. For the continuous quantitative variables, the Wilcoxon rank sum test was used. We used logistic regression to estimate the odds for developing T2DM among PLWHIV (Additional file 2). We used Cramer’s V post-test for strength of association following Chi square test in Table 3. We performed a receiver operator curve (ROC) using risk scores to predict T2DM (Additional file 3).

**Table 2:** Prevalence of T2DM and associated factors

Variables	N = 234	Non-diabetic n = 226 (96.9%)	T2DM n = 8 (3.4%)	p value
Age, median years (IQR)	44 (36, 52)	43 (37, 53)	55 (49, 59)	*0.016*
Age category (years)				
18–35	57 (24.4)	57 (25.2)	0 (0.0)	*0.018*
36–45	73 (32.3)	73 (32.3)	0 (0.0)	
46–55	66 (28.2)	60 (26.5)	6 (9.1)	
56–65	33 (14.1)	31 (13.7)	2 (6.1)	
66–90	5 (2.1)	5 (2.2)	0 (0.0)	
Gender, n (%)				
Female	155 (66.2)	149 (96.1)	6 (3.9)	0.720
Male	79 (33.8)	77 (97.5)	2 (2.5)	
BMI, median (IQR) kg/m^2^	22.4 (19.7, 27.1)	22.4 (19.8, 26.4)	27.3 (22.8, 32.2)	*0.040*
Waist circumference (cm)	80 (72, 90)	79 (71, 90)	90 (84, 103)	0.081
Duration on ART, median months (IQR), n = 233	96 (48, 132)	102 (72, 132)	132 (54, 168)	0.671
ART regimen, n = 206				
TDF/3TC/EFV	148 (71.8)	142 (95.9)	6 (4.1)	0.877
TDF/3TC/NVP	19 (9.2)	19 (100.0)	0 (0.0)	
TDF/3TC/LPV/r	15 (7.3)	15 (100.0)	0 (0.0)	
ABC/3TC/NVP	1 (0.5)	1 (100.0)	0 (0.0)	
ABC/3TC/EFV	5 (2.4)	5 (100.0)	0 (0.0)	
AZT/3TC/LPV/r	7 (3.4)	7 (100.0)	0 (0.0)	
AZT/3TC/NVP	11 (5.3)	11 (100.0)	0 (0.0)	
CD4+ absolute count median (IQR) cells/µl, n = 148	473 (360, 644)	460 (378, 653)	631 (378, 681)	0.356
HIV RNA viral load (copies/ml), n = 192	33 (20, 308)	20.5 (20, 379)	20 (20, 605)	0.489
Hypertension				
No	197 (84.2)	191 (97.0)	6 (3.0)	0.616
Yes	37 (15.8)	35 (94.6)	2 (5.4)	
Minutes of weekly vigorous activity	0 (0, 90)	0 (0, 120)	0 (0, 60)	0.521
Minutes of weekly moderate activity	60 (9, 123)	40 (0, 180)	60 (37, 150)	0.573
Minutes of weekly walking activity	60 (30, 120)	60 (20, 120)	12 (5, 75)	0.056
Days of weekly vegetable intake	7 (7, 7)	7 (6, 7)		0.147
Days of weekly fruit intake	1 (0, 3)	1 (0, 3)	0 (1, 3)	0.098
Minutes spend sited every weekend	300 (120, 480)	300 (150, 397)	360 (240, 750)	0.402
Minutes spend sited every weekday	180 (120, 360)	210 (120, 360)	300 (150, 450)	0.803
Fasting blood sugar	5.2 (4.7, 5.8)	5.0 (4.6, 5.5)	7.2 (4.4, 9.9)	*0.001*
Risk scores	5 (2, 9)	5 (2, 8)	16 (14, 19)	*< 0.001*

*n* number of participants, *%* percentage, *BMI* body mass index, *T2DM* type 2 diabetes mellitus, *IQR* interquartile range, *TDF* tenofovir disoproxil fumarate, *3TC* lamivudine, *EFV* efavirenz, *NVP* nevirapine, *LPV/r* lopinavir/ritonavir, *ABC* abacavir, *AZT* azidothymidine, also called zidovudine

p values less than 0.05 are shown in italic

**Table 3:** Strength of association between risk category and T2DM

Risk category (scores)	Non-diabetic n = (%)	T2DM, n = (%)	p value
Several categories			
Low risk (< 7)	135 (59.7)	0 (0.0)	*< 0.001*
Slightly elevated risk (7–11)	67 (29.6)	1 (12.5)	
Moderate (12–14)	17 (7.5)	2 (25.0)	
High (15–20)	7 (3.1)	5 (62%)	
Cramer’s V	0.513		< 0.001
Binary category			
Low risk (< 7 scores)	135 (59.7)	0 (0.0)	*0.001*
Increased risk (≥ 7 scores)	91 (40.3)	8 (100.0)	
Cramer’s V	0.220		0.001

*n* number of participants, *%* percentage, *T2DM* type 2 diabetes mellitus; Fisher’s exact test used; column percentage shown

p values less than 0.05 are shown in italic

### Results

#### Basic characteristics of study participants

The study was composed of 234 participants. Participants (Additional file 1), were younger than 45 years (54%), had BMI between 18.5 and 25 (53%), ate vegetables/fruit daily (79%), had no first-degree family members with diabetes mellitus (77%), had no history of taking BP medications (84%), had a waist circumference between 60 and 94 cm (for men) or 60 and 80 cm (for women) (59%), had no history of high plasma glucose (97%) and were in the low risk category (58%). Forty six percent (46%) had daily physical activity. The median risk score in the population was 5.

Age, gender, BMI, waist circumference, hypertension status (p < 0.001), and days of weekly vegetable intake (p = 0.028) were the only factors significantly associated with increased risk for developing T2DM based on risk scores (Table 1). However, when adjusted in multivariate regression (Additional file 2) for all factors that were significant in univariate logistic regression, only age remained significant (p = 0.013).

The prevalence of diabetes mellitus type 2 in the study population was 3% (Table 2) and only age, age category, BMI, fasting blood sugar and risk scores were associated with T2DM.

As shown in Table 3, where risk scores are categorized into several categories and also dichotomized, both were associated with T2DM. However, the diabetes risk category with several levels (Cramer’s v, 0.513) was the best fit model associated with T2DM compared to dichotomized risk (Cramer’s v, 0.220) as shown by using Cramer’s v post-test for strength of association.

#### Risk scores to predict type 2 diabetes mellitus on receiver operator curve (ROC)

Receiver operator curve (Additional file 3) was employed to test the diagnostic role of using risk scores to predict T2DM. The area under the curve was 0.952, standard error of 0.025 (0.90, 1.00 95% CI, p < 0.001). This means a diabetes patient will have higher test scores than 95% of healthy individuals on average. The best predictive score was 11.5 yielding 88% sensitivity and 89% specificity.

### Discussion

In this study, PLWHIV had median risk score of 5 (2, 9, IQR) and only age remained significantly associated with T2DM after adjusting for other variables (p = 0.013) (Table 2 and Additional file 2). The prevalence of T2DM in the study population was 3%. This study demonstrated that diabetes risk scores can be used as a surrogate for identifying patients with or at risk of developing T2DM as shown in Table 3 and Additional file 1.

From our study, we classified PLWHIV according to the risk categories for developing T2DM in 10 years as follows: 58%—low risk, 29%—slightly elevated risk, 8%—moderate risk and 5%—high risk. On the dichotomized scale (< 7 versus ≥ 7 scores), 42% were at risk of developing T2DM, slightly higher than the report from Cameroon were they reported 31% [11]. However, we can not ascertain from this study, the validity of the estimated risk surfice to say several studies [2–4] confirm that HIV positive individuals are more likely to develop diabetes mellitus compared to the general population but some studies have shown no differences between the two groups [18] though limited by the high heterogeneity, moderate-to-high risk of bias, and small number of studies included in the systematic review [18]. The increased risk may be due to the HIV infection and ART [6] that results in metabolic derangements [2, 4]. However, the scope of our study could not ascertain this and all study participants were on cART with no specific cART regimen associated with T2DM.

#### Risk scores and T2DM

We demonstrated from this study, that diabetes risk scores can be employed to identify high risk individuals for developing T2DM. When risk was dichotomized (Table 1), 42% versus 58% had increased risk for developing T2DM. However, using several categories as they appear in the diabetes risk assessment form, only 5% were identified to be at high risk for developing T2DM (Table 3). This percentage is higher but close to the actual 3.4% prevalence of T2DM in our study population (Table 3). Similarly, another study conducted in the copperbelt province of Zambia reported a 5% prevalence of diabetes in treated PLWHIV [12]. Furthermore, this study also demonstrated that the several risk categories as used in the diabetes risk assessment form are better to predict risk than the dichotomized category going by the strength of association using Cramer’s V post-test (Table 3). To strengthen the concept of using risk scores to identify high risk individuals for developing T2DM, we employed a ROC analysis (Additional file 3) which demonstrated that T2DM patients will have higher test scores than 95% of healthy individuals and the best predictive score was 11.5. However, further studies, more especially epidemiological studies are warrantable to explore this area.

### Conclusion

Based on the dichotomized risk threshold of 7 scores, about 42% of PLWHIV were at risk for developing T2DM in 10 years. However, only about 5% were categorized to be at high risk using scores of 15–20. The actual prevalence of T2DM in the population was 3.4%.

#### Recommendation

We recommend ART clinics to embed diabetes assessments in their HIV routine care. However, caution should be taken when identifying those individuals who really are at high risk of developing T2DM as several risk assessment tools are obtaining [15] and populations may differ. Moreover, in the neighboring country of Botswana, a study revealed that the FINDRISC was only modestly effective in predicting undiagnosed diabetes among outpatients [19].

## Limitations

Type 2 diabetes mellitus is a very complex, multi-factorial disease with very high phenotypic variation dependant upon multiple anthropometric, biochemical and genetic factors which vary by population. A larger sample size is required with a wide range of variables to understand this area. The current study was limited by this. However, the data generated from this study remains important within its context.

## Additional files

**Additional file 1.** Participant distribution characteristics based on Diabetes risk score form.

**Additional file 2.** Risk for developing T2DM among PLWHIV in univariate and multivariate binary logistic regression.

**Additional file 3.** Risk scores to predict type 2 diabetes mellitus on receiver operator curve (ROC).

## Abbreviations

BMI: body mass index
BP: blood pressure
cART: combinational antiretroviral therapy
CRS: Cambridge Risk Score
FINDRISC: Finnish Diabetes Risk Score
HIV: human immune deficiency virus
IDF: international diabetes federation
IQR: interquartile range
LCH: Livingstone Central Hospital
PLWHIV: people living with HIV
SPSS: statistical package for social sciences
STROBE: Strengthening the Reporting of Observational Studies in Epidemiology
T2DM: type 2 diabetes mellitus
UNZABREC: University of Zambia Research Ethics Committee
