# Supplementary material for: Type 2 diabetes mellitus prevalence and risk scores in treated PLWHIV: a cross-sectional preliminary study
Source: BMC Res Notes. 2019 Mar 15;12:145. doi: 10.1186/s13104-019-4183-6 (PMC6420761; doi:10.1186/s13104-019-4183-6)
Supplement: Supplementary file 1 — Additional file 1. Participant distribution characteristics based on Diabetes risk score form. [file 13104_2019_4183_MOESM1_ESM.docx]

**Additional File: 1**

Table S1. Participant distribution characteristics based on Diabetes risk score form

| **Variable** | **n (%)** | **Variable** | **n (%)** |
| --- | --- | --- | --- |
| **Age category (years)** |  | **Risk category** |  |
| 18 to 44 | 126 (53.8) | Low | 135 (57.7) |
| 45 to 54 | 63 (26.9) | Slightly elevated | 68 (29.1) |
| 55 to 64 | 38 (16.2) | moderate | 19 (8.1) |
| 65-90 | 7 (3.0) | high | 12 (5.1) |
|  |  | Very high | 0 (0.0) |
| **BMI (kg/m^2^)** | |  |  |
| 14 -18.4 | 31 (13.2) | **Waist circumference (cm)** |  |
| 18.5 -24.9 | 125 (53.4) | 60 - 94 men; 60-80 women | 139 (59.4) |
| 25.0 to 30.0 | 47 (20.1) | 94-102 men; 80-88 women | 40 (17.1) |
| 30.1 – 51.0 | 31 (13.2) | 103-120 men; 88-120 women | 55 (23.5) |
| **Daily physical activity** |  | **Ever taken BP medications** |  |
| Yes | 108 (46.2) | Yes | 37 (15.8) |
| No | 126 (53.8) | No | 197 (84.2) |
| **Daily Vegetable or Fruit intake** |  | **Ever had high plasma Glucose** |  |
| Yes | 185 (79.1) | Yes | 8 (3.4) |
| No | 49 (20.9) | No | 226 (96.6) |
| **Family history of Diabetes** |  | **Risk score (median, IQR)** | 5 (2, 9) |
| No first degree family members with diabetes | 179 (76.5) |  |  |
| Grandparent, aunt, uncle or first cousin with diabetes mellitus | 4 (1.7) |  |  |
| Parent, brother, sister or own child with diabetes mellitus | 51 (21.8) | **Total** | **234** |

n, number of participants; %, percentage; BMI, body mass index; IQR, interquartile range; BP, blood pressure

The categorization of the risk scores and variables were as follows: Low risk: < 7 scores, Slightly elevated risk: 7-11 scores, moderate risk: 12-14 scores, high risk: 15-20 scores and very high risk: >20 scores. The ‘risk categories’ denote a 10-year estimated risk of developing T2DM in this study. *‘*Daily physical activity’ where ‘yes’ meant that the participant was involved in moderate and/or vigorous physical activity every day of the week; ‘Daily vegetable or fruit intake’ where ‘yes’ meant that the participant ate vegetable and/or fruit every day of the week; ‘waist circumference’ (units used were centimeters (cm)) where the cut-off denoting normal for men was <94 and <80 for women whereas 94-102 and 80-88 for men and women respectively was considered moderately large while >102 (men) and >88 (women) was considered larger; ‘ever taken blood pressure (BP) medications’ were to identify those with hypertension; ‘ever had high plasma glucose’ were to identify any participants that had ever recorded a fasting plasma glucose equal to or higher than 6.9 mmol/l or random plasma glucose equal to or higher than 11.1mmol/l.
