# Supplementary material for: Type 2 diabetes mellitus prevalence and risk scores in treated PLWHIV: a cross-sectional preliminary study
Source: BMC Res Notes. 2019 Mar 15;12:145. doi: 10.1186/s13104-019-4183-6 (PMC6420761; doi:10.1186/s13104-019-4183-6)
Supplement: Supplementary file 2 — Additional file 2. Risk for developing T2DM among PLWHIV in univariate and multivariate binary logistic regression. [file 13104_2019_4183_MOESM2_ESM.docx]

Additional file 2

**Table S2:** Risk for developing T2DM among PLWHIV in Univariate and Multivariate binary logistic regression

| **Variable** | **Odds Ratio (OR) (95%CI)** | **p-value** | **Adjusted Odds Ratio AOR (95%CI)** | **p-value** |
| --- | --- | --- | --- | --- |
| Age, *years* | 1.06 (1.03, 1.08) | **<0.001** | 1.05 (1.01, 1.08) | **0.013** |
| Gender |  |  |  |  |
| Female | 1.00 |  | 1.00 |  |
| Male | 0.33 (0.18, 0.59) | **<0.001** | 0.43 (0.18, 1.07) | 0.071 |
| BMI, kg/m^2^ | 1.33 (1.23, 1.45) | **<0.001** | 1.15 (0.99, 1.33) | 0.055 |
| Waist circumference (cm), | 1.12 (1.09, 1.16) | **<0.001** | 1.05 (0.99, 1.12) | 0.091 |
| Hypertension |  |  |  |  |
| No | 1.00 |  | 1.00 |  |
| Yes | 0.18 (0.08, 0.41) | **<0.001** | 2.51 (0.84, 7.55) | 0.101 |
| Minutes of weekly vigorous activity | 0.99 (0.99, 1.00) | **0.019** | 0.99 (0.99, 1.00) | 0.225 |
| Days of weekly vegetable intake | 0.85 (0.72, 0.99) | **0.036** | 0.82 (0.67, 1.01) | 0.064 |
| Fasting blood sugar | 1.44 (1.03, 2.00) | **0.032** | 1.13 (0.77, 1.66) | 0.546 |

BMI, body mass index; PLWHIV, people living with HIV; T2DM, type 2 diabetes mellitus. Significant associations are shown by bold p values. Outcome variable used is ‘risk’ dichotomized, the rest are independent variables.
