# Supplementary material for: Type 2 diabetes mellitus prevalence and risk scores in treated PLWHIV: a cross-sectional preliminary study
Source: BMC Res Notes. 2019 Mar 15;12:145. doi: 10.1186/s13104-019-4183-6 (PMC6420761; doi:10.1186/s13104-019-4183-6)
Supplement: Supplementary file 3 — Additional file 3. Risk scores to predict type 2 diabetes mellitus on receiver operator curve (ROC). [file 13104_2019_4183_MOESM3_ESM.docx]

**Additional file 3**


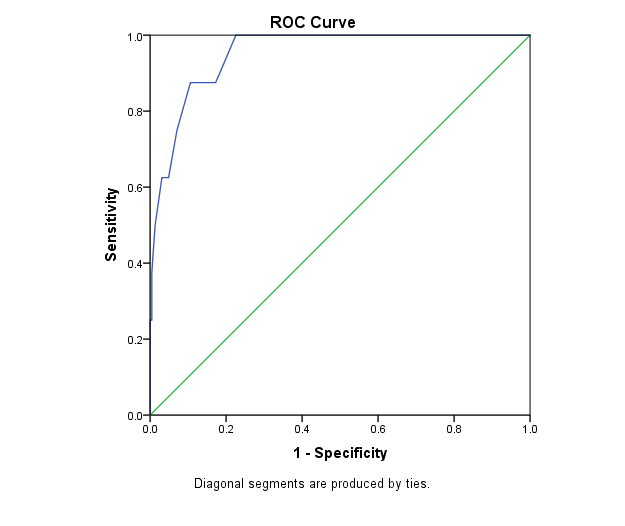


Figure S1. Risk scores to predict type 2 diabetes mellitus on receiver operator curve (ROC)

Area under the curve 0.952, standard error of 0.025 (0.90, 1.00 95%CI, P <0.001). This means on average; a diabetes patient will have higher test scores than 95% of healthy individuals.

The best predictive score was 11.5 yielding 88% sensitivity and 89% specificity.
